# Cytomegalovirus (CMV)-Specific Cell-Mediated Immunity for Prediction of Postprophylaxis CMV Disease in a Phase 3 Trial of Letermovir Versus Valganciclovir Prophylaxis in Donor CMV-Seropositive/Recipient CMV-Seronegative Kidney Transplant Recipients

**DOI:** 10.1093/cid/ciaf632

**Published:** 2025-12-01

**Authors:** Ajit P Limaye, Marta Crespo, Nassim Kamar, Atul Humar, Robert P Carroll, Marcus R Pereira, Natalya Broyde, Weiwen Wang, Barbara Haber

**Affiliations:** Division of Infectious Diseases, Transplant and Immunocompromised Host Infectious Disease Program, University of California, San Francisco, San Francisco, California, USA; Nephrology Department and Kidney Transplant Unit, Hospital del Mar, Nephropathy Research Group, Hospital del Mar Research Institute, Universitat Pompeu Fabra, Barcelona, Spain; Department of Nephrology and Organ Transplantation, Toulouse Rangueil University Hospital, INSERM UMR 1291, Toulouse Institute for Infectious and Inflammatory Diseases (Infinity), Paul Sabatier University, Toulouse, France; Division of Infectious Diseases, Department of Medicine, Ajmera Transplant Center, University Health Network, University of Toronto, Toronto, Ontario, Canada; Transplantation Immunogenetics Service, LifeBlood, Adelaide, South Australia, Australia; Division of Infectious Disease, Department of Medicine, Columbia University College of Physicians and Surgeons, New York, New York, USA; Merck Research Laboratories, Merck & Co., Inc., Rahway, New Jersey, USA; Merck Research Laboratories, Merck & Co., Inc., Rahway, New Jersey, USA; Merck Research Laboratories, Merck & Co., Inc., Rahway, New Jersey, USA

**Keywords:** cytomegalovirus, cell-mediated immunity, QuantiFERON-CMV, kidney transplant, CMV donor-seropositive recipient-seronegative

## Abstract

**Background:**

QuantiFERON-cytomegalovirus (QFT-CMV) is a standardized assay for the evaluation of cytomegalovirus-specific cell-mediated immunity (CMV-CMI).

**Objectives:**

The purpose of this study was to assess the evolution of CMV-CMI posttransplant and the clinical utility of QFT-CMV for the prediction of postprophylaxis CMV disease in CMV-seronegative recipients of CMV-seropositive donor kidneys (D+R− KTRs).

**Methods:**

601 adult CMV D+R− kidney transplant recipients (KTRs) received letermovir or valganciclovir prophylaxis for 28 weeks in a phase 3, double-blind, multicenter trial (ClinicalTrials.gov NCT03443869). QFT-CMV was performed at a central laboratory by masked personnel at transplant, 12, 28, and 52 weeks posttransplant. Investigators assessed CMV disease through week 52. Sensitivity, specificity, and positive and negative predictive values (PPV, NPV) at week 28 were used to evaluate the clinical utility of QFT-CMV.

**Results:**

Positive QFT-CMV results (pooled) were detected: baseline–1.2%, week 12–2.6%, week 28–7.7%, and week 52–28.9%. The distribution of positive results with letermovir and valganciclovir was comparable, except at week 28 (letermovir 2.2% and valganciclovir 12.8%). Postprophylaxis CMV disease by week 52 occurred in 18.3% (84/460) of evaluable participants: 12.5% (4/32) of participants with a positive, 17.5% (66/377) with a negative, and 27.5% (14/51) with an indeterminate result at week 28 (p = not significant for all comparisons). QFT-CMV sensitivity, specificity, PPV, and NPV were 8.3%, 94.3%, 87.5%, and 17.5%, and were similar when pooling indeterminate and negative results and within the letermovir and valganciclovir study arms.

**Conclusions:**

Among adult CMV high-risk D+R− KTRs who received prophylaxis, CMV-CMI by QFT-CMV increased over time. The result at the end of prophylaxis had limited clinical utility for identifying the risk for subsequent CMV disease.

## Teaser

Among adult cytomegalovirus (CMV) donor-seropositive/recipient-seronegative kidney transplant recipients who received antiviral prophylaxis with letermovir or valganciclovir in a large, randomized, phase 3 trial, QuantiFERON-CMV had limited clinical utility for identifying participants at high-risk for subsequent CMV disease.

**Clinical Trials Registration**. ClinicalTrials.gov: NCT03443869, EudraCT: 2017-001055-30

**URL**: https://clinicaltrials.gov/ct2/show/NCT03443869?term=NCT03443869&draw=2&rank=1

## Graphical Abstract

**Graphical Abstract:**
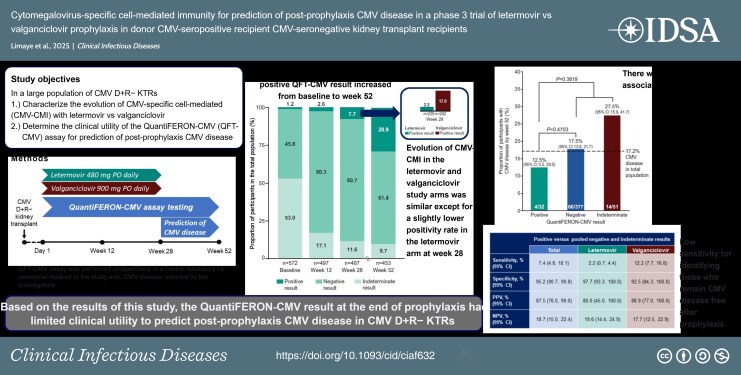

**(See the Editorial Commentary by Natori and Sester on pages e325–7.)**

Cytomegalovirus (CMV) remains a major opportunistic pathogen in kidney transplant recipients (KTRs), with the greatest risk of CMV disease in CMV-seronegative recipients (R−) who receive kidneys from CMV-seropositive donors (D+) [1, 2]. Antiviral prophylaxis is generally effective for CMV disease prevention among those who tolerate it during the time that it is given in CMV D+R− KTRs [3, 4]. Approximately 15–30% of CMV D+R− KTRs develop postprophylaxis CMV disease that is not well predicted by standard clinical factors [4–9]. In addition to direct attributable morbidity, mortality, and increased costs, postprophylaxis CMV disease is associated with an increased risk for allograft failure and mortality during longer-term follow-up [9].

CMV-specific cellular (T-cell) immunity (CMV-CMI) plays a major role in protection against CMV disease in solid organ transplant (SOT) recipients, although humoral immunity may also be important [10–13]. Several standardized and commercially available CMV-CMI assays have been studied for predicting postprophylaxis CMV disease [14]. These studies have generally reported better assay operating characteristics and clinical utility in CMV R+ compared to D+R− groups [15–20]. However, studies of CMV-CMI, specifically in the CMV D+R− group, have been limited by relatively small sample sizes, mixed organ transplant populations, and/or nonstandard definitions for CMV outcomes (Supplementary Table S1) [15–17, 21–26].

To address the limitations of prior studies, we assessed serial CMV-CMI by QuantiFERON-CMV (QFT-CMV) and its association with postprophylaxis CMV disease among participants in a large, multicenter, multinational trial of letermovir versus valganciclovir prophylaxis in CMV D+R− KTRs. The objectives of this study were to (1) characterize the evolution of CMV-CMI posttransplant and (2) determine the clinical utility of the QFT-CMV assay for the prediction of postprophylaxis CMV disease in CMV D+R− KTRs, in the pooled population and separately in the letermovir and valganciclovir arms.

## METHODS

### Study Design and Participants

In a phase 3, double-blind, double-dummy, multicenter trial, adult (≥18 years old) CMV D+R− KTRs were randomized 1:1 (stratified by receipt of lymphocyte-depleting induction immunosuppression) to prophylaxis with letermovir 480 mg orally once daily or valganciclovir 900 mg orally once daily for 28 weeks (∼200 days) posttransplant (MK-8228-002, ClinicalTrials.gov NCT03443869). Participants in the letermovir arm received a matching valganciclovir placebo orally once daily and acyclovir 400 mg orally twice daily (added for prevention of herpes simplex virus [HSV] and varicella zoster virus [VZV]). Participants in the valganciclovir (providing prevention for CMV, HSV, and VZV) arm received a matching letermovir placebo orally once daily and a matching acyclovir placebo orally twice daily. Valganciclovir and acyclovir were dosed as recommended in prescribing information and guidelines based on Cockcroft-Gault creatinine clearance [14, 27, 28]. The study was conducted in accordance with principles of Good Clinical Practice and was approved by the appropriate institutional review boards and regulatory agencies. All participants provided written informed consent. The study took place between May 2018—April 2021 at 94 global sites. Complete details of the study design are available in the primary clinical trial manuscript [3].

Investigators monitored participants for the development of CMV disease for 52 weeks posttransplant. Monitoring of CMV DNA for the clinical management of participants was performed locally at the investigators' discretion. Treatment for suspected CMV disease was also at the investigators' clinical discretion based on local standards and guidelines (i.e., clinical, laboratory, radiographic, and/or histopathological data).

Investigators were instructed to collect plasma samples for quantitative CMV DNA at prespecified study visits and upon suspicion of CMV infection or disease for processing at a central laboratory (Roche COBAS® AmpliPrep/COBAS TaqMan® assay; lower limit of quantification 137 IU/mL). Central laboratory results for quantitative CMV DNA were not shared with investigators.

**Figure 1.**
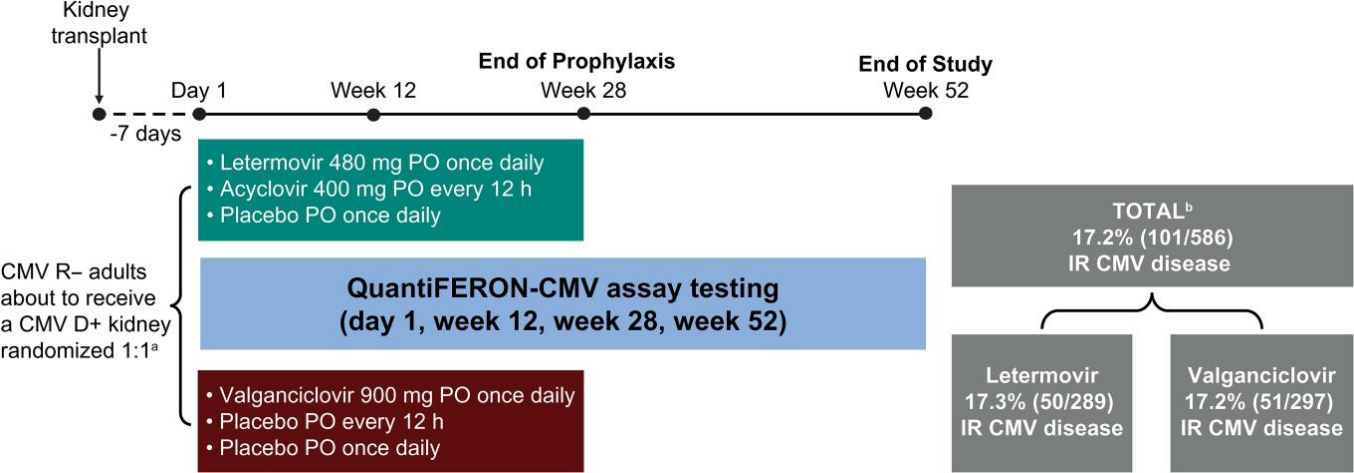
Study design. ^a^Stratified by receipt of ≥1 of the following lymphocyte-depleting agent(s) at the time of transplant: horse-derived or rabbit-derived antithymocyte globulin, alemtuzumab, or muromonab CD3. ^b^Participants had to receive ≥1 dose of study regimen and be CMV D+R– and negative for CMV DNAemia on day 1 to be included; 3 participants were excluded from the letermovir arm (1 was CMV D+R+ and 2 had CMV DNAemia on day 1). Abbreviations: CMV, cytomegalovirus; D+, seropositive donor; IR, investigator–reported; R–, seronegative recipient.

The QFT-CMV assay was performed prospectively in a central laboratory by personnel masked to the study arm and interpreted per manufacturer recommendations (Supplementary Table S2) [29] on samples collected at baseline (day 1 of prophylaxis; prophylaxis initiation within 7 days posttransplant), and at 12, 28, and 52 weeks posttransplant (Figure 1). Central laboratory QFT-CMV assay results were not shared with site investigators.

### Outcomes

Outcomes were assessed in all randomized CMV D+R− KTRs who received ≥1 dose of the study regimen and had no detectable quantifiable CMV DNA as measured by the central laboratory at baseline (full analysis set). QFT-CMV assay results were assessed at baseline, at weeks 12 and 28 (end of prophylaxis), and postprophylaxis (at 52 weeks posttransplant). As muted lymphocyte activity or lymphopenia may be associated with an indeterminate QFT-CMV result [29], the effect of lymphocyte-depleting induction immunosuppression on the QFT-CMV result was explored by assessing the QFT-CMV result at baseline and the proportion of participants who received ≥1 lymphocyte-depleting agent(s). The clinical utility of the QFT-CMV assay at week 28 was determined by assessing the association of the QFT-CMV result at week 28 (end of prophylaxis) with investigator-reported CMV disease between weeks 28 and 52 posttransplant. A sensitivity analysis was performed with CMV disease confirmed by an external masked clinical adjudication committee. For the clinical utility analyses, participants who had developed CMV disease prior to week 28 were excluded. A supportive analysis evaluated week 52 QFT-CMV results and prior CMV events (investigator-reported CMV disease, quantifiable CMV DNAemia, and investigator-reported CMV disease and/or quantifiable CMV DNAemia) from day 1 through week 52.

### Statistical Analyses

Descriptive statistics, with corresponding 95% confidence intervals (CIs) as appropriate, were used to characterize baseline demographics/characteristics and outcomes. To investigate if sex, age, race, region, lymphocyte-depleting induction, and donor type were associated with the week 28 QFT-CMV result, the difference (95% CI) between the proportion of participants with a positive versus a negative or indeterminate result was determined. A 2-sided chi-square test was used to evaluate the association between the QFT-CMV result at week 28 (either positive versus negative, or positive versus negative and indeterminate combined) with CMV disease by week 52.

The sensitivity, specificity, positive predictive value (PPV), and negative predictive value (NPV) of the QFT-CMV result at 28 weeks posttransplant (the time of antiviral prophylaxis discontinuation) were assessed for the prediction of subsequent CMV disease by 52 weeks posttransplant. In this analysis, sensitivity expressed the likelihood that a participant with a positive QFT-CMV result would remain CMV disease-free. Specificity measured whether a negative (and indeterminate) result indicated that a participant would develop CMV disease. PPV expressed the probability of remaining CMV disease-free postprophylaxis with a positive QFT-CMV result. NPV expressed the probability of developing postprophylaxis CMV disease with a negative (and indeterminate) QFT-CMV result.

## RESULTS

### Study Population

**Figure 2.**
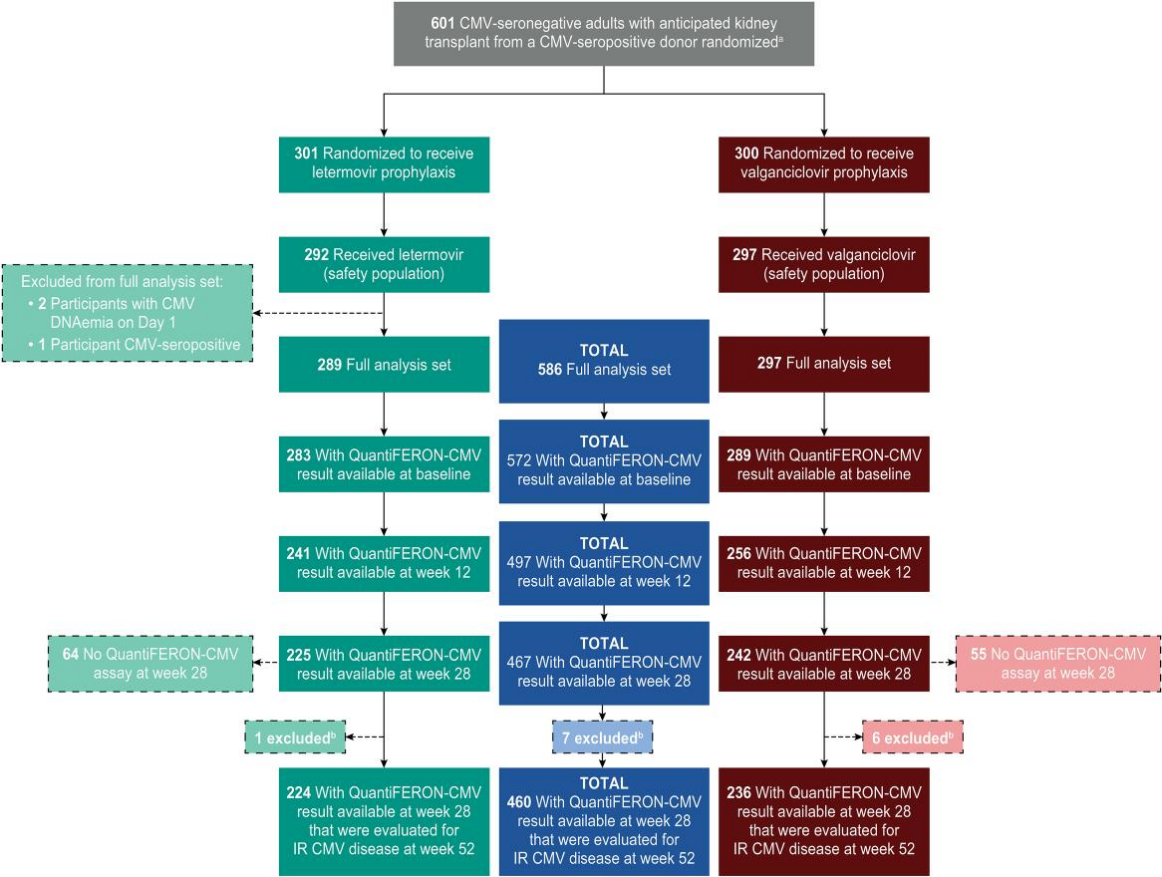
Participant disposition.^a^Stratified by receipt of ≥1 of the following lymphocyte-depleting agent(s) at the time of transplant: horse-derived or rabbit-derived antithymocyte globulin, alemtuzumab, or muromonab CD3. ^b^Participants excluded because they developed IR CMV disease prior to week 28. Abbreviations: CMV, cytomegalovirus; IR, investigator-reported.

The full analysis set consisted of 586 CMV D+R− KTRs. In the full analysis set, 72% of participants were male, the mean age was 49.6 years, and 46% received lymphocyte-depleting induction immunosuppression. Baseline demographics and characteristics were comparable in the letermovir (n = 289) and valganciclovir (n = 297) arms (Table 1). A total of 470 participants completed prophylaxis (246 in the letermovir arm and 224 in the valganciclovir arm) in the full analysis set. Investigator-reported CMV disease occurred in 17.2% (101/586) of participants in the full analysis set at week 52 [3]. Participant disposition from baseline through week 28 for this study is depicted in Figure 2.

**Table 1.** Demographics and Characteristics of Participants in the Full Analysis Set at Baseline

	Letermovir (n = 289)	Valganciclovir (n = 297)	Total (N = 586)
Sex, male, n (%)	210 (72.7)	209 (70.4)	419 (71.5)
Age, mean (SD), years	49.6 (14.6)	49.6 (15.1)	49.6 (14.8)
Race, n (%)			
Asian	4 (1.4)	10 (3.4)	14 (2.4)
Black or African American	21 (7.3)	33 (11.1)	54 (9.2)
White	250 (86.5)	243 (81.8)	496 (84.2)
Other	14 (4.8)	11 (3.7)	25 (4.3)
Use of lymphocyte-depleting induction immunosuppression, n (%)^a^	131 (45.3)	138 (46.5)	272 (46.2)
Donor type, n (%)			
Living	120 (41.5)	115 (38.7)	235 (40.1)
Deceased	169 (58.5)	182 (61.3)	351 (59.9)

^a^Stratified by receipt of ≥1 of the following lymphocyte-depleting agent(s) at the time of transplant: horse-derived or rabbit-derived antithymocyte globulin, alemtuzumab, or muromonab CD3.

### Evolution of QuantiFERON-CMV Assay Results During and Postprophylaxis

**Figure 3.**
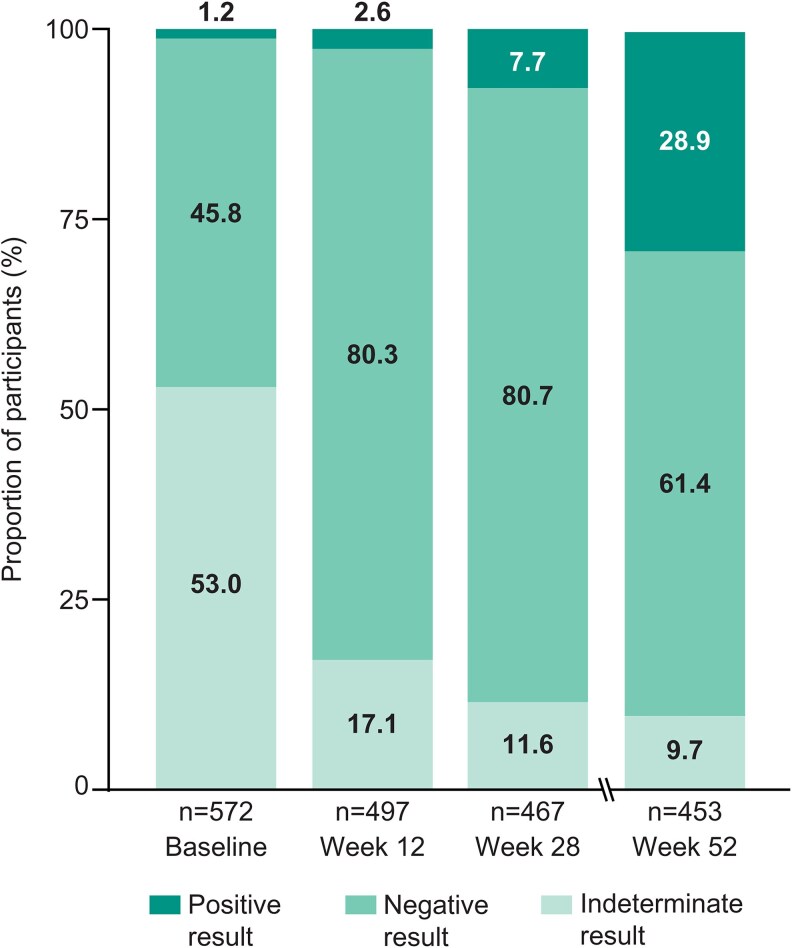
QuantiFERON-CMV results over time in the total population.

**Figure 4.**
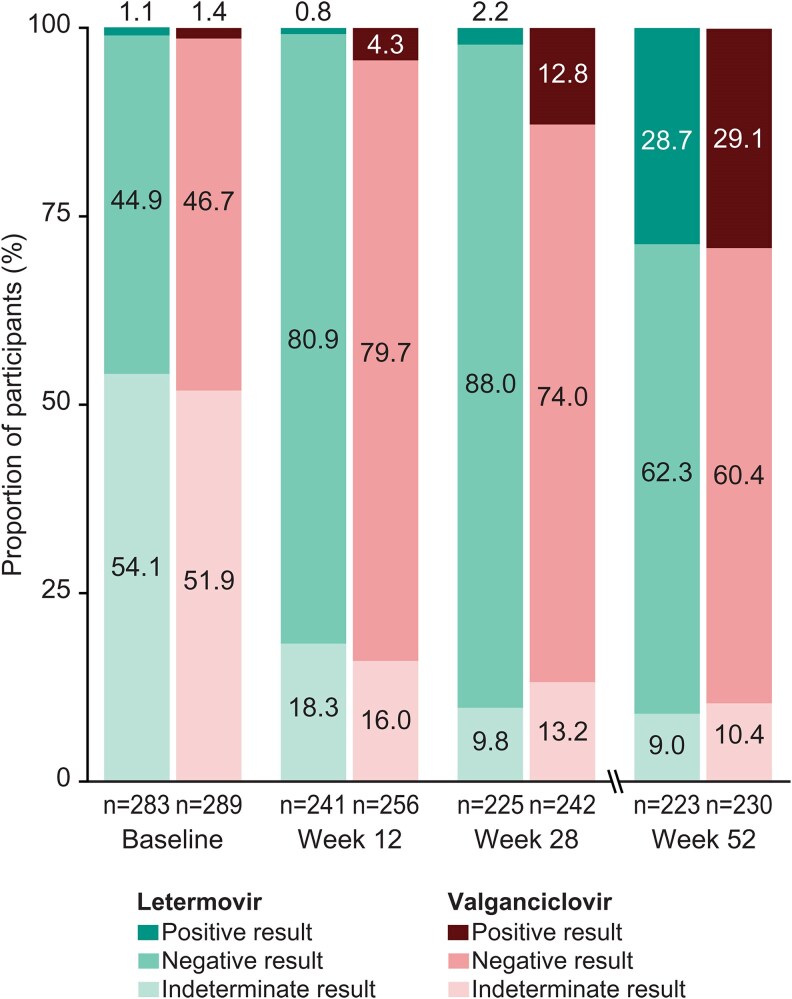
QuantiFERON-CMV results over time by study arm.

The proportion of participants with a positive QFT-CMV result increased from baseline (1.2%) to week 28 (7.7%). By week 52, 28.9% of participants in the total population had a positive result. The majority of participants had a negative QFT-CMV result at week 28 (80.7%) and week 52 (61.4%) posttransplant. A high proportion of participants had an indeterminate QFT-CMV result (53.0%) at baseline, but at weeks 28 and 52, the proportion with an indeterminate result had decreased to approximately 10% (Figure 3). Participants who received lymphocyte-depleting induction immunosuppression were 2–3 times more likely to have an indeterminate QFT-CMV result compared to participants with a positive or negative result (Supplementary Table S3), and the proportion of participants with an indeterminate result diminished over time. The distribution of QFT-CMV results over time in the letermovir and valganciclovir arms was generally comparable to those observed in the total pooled population. However, at week 28, the proportion with a positive result was lower in the letermovir (2.2%, 95% CI 0.7-5.1) versus valganciclovir (12.8%, 95% CI 8.9-17.7) arm (Figure 4).

### Factors Associated With Week 28 QuantiFERON-CMV Assay Results

QFT-CMV results were available for 80% (467/586) of participants in the full analysis set at week 28 (Figure 2). Participants with (467) and without (119) a QFT-CMV result at week 28 had comparable baseline demographics (Supplementary Table S4). Baseline demographics and characteristics were generally similar among participants with positive, negative, and indeterminate assay results at week 28 (Supplementary Table S5). Sex, age, race, region, lymphocyte-depleting induction, and donor type were not associated with the QFT-CMV result (Supplementary Figure S1). A total of 36 participants had a positive QFT-CMV at week 28. Eleven (30.6%) of these participants (letermovir 1, valganciclovir 10) had CMV DNAemia, and 25 (69.4%) did not have CMV DNAemia (letermovir 4, valganciclovir 21) through week 28. The letermovir participant with the positive QFT-CMV result at week 28 had discontinued letermovir prophylaxis early (day 25) due to an adverse event, began valganciclovir treatment (day 48), and the CMV DNAemia was detected during valganciclovir treatment (day 112). Of the 10 participants in the valganciclovir arm who had CMV DNAemia prior to week 28 and a positive QFT-CMV at week 28, 4 had CMV DNAemia after premature discontinuation of prophylaxis due to adverse events or physician decision, and 6 had CMV DNAemia during prophylaxis (i.e. breakthroughs).

### Association of the Week 28 QuantiFERON-CMV Result With Postprophylaxis Cytomegalovirus Disease Through Week 52 Posttransplant

**Figure 5.**
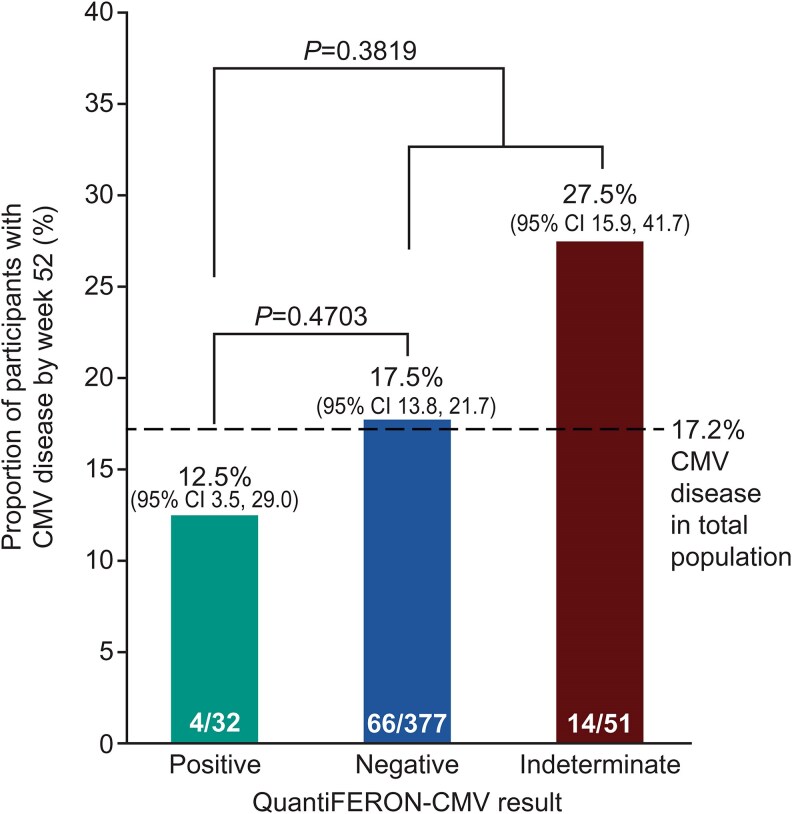
QuantiFERON-CMV assay results at week 28 in the total population and investigator-reported postprophylaxis CMV disease by week 52. Chi square test with two-tailed *P*-value for positive versus negative results and positive versus pooled negative and indeterminate results.

Of the 467 participants with a QFT-CMV result available at week 28, 7 were excluded because they had already developed investigator-reported CMV disease prior to week 28. Of the remaining 460 participants with a QFT-CMV result at week 28, 84 (18.3%) subsequently developed investigator-reported CMV disease by week 52. CMV disease by week 52 occurred in 12.5% (4/32) of participants with a positive QFT-CMV result, 17.5% (66/377) with a negative QFT-CMV result, and 27.5% (14/51) of participants with an indeterminate QFT-CMV result. There was no significant association between QFT-CMV result at week 28 (positive versus negative result or positive versus negative and indeterminate result combined) and subsequent CMV disease (Figure 5). In a sensitivity analysis restricted to those with committee-confirmed CMV disease, the results were similar, with the proportion of participants with postprophylaxis CMV disease at week 52 with a positive, negative, and indeterminate QFT-CMV result at week 28 was 9.4% (3/32), 11.7% (44/377), and 13.0% (7/54), respectively (Supplementary Figure S2).

Assay sensitivity, specificity, PPV, and NPV for investigator-reported CMV disease postprophylaxis were 8.3% (95% CI 5.3 to 11.2), 94.3% (95% CI 88.9 to 99.7), 87.5% (95% CI 76.0 to 99.0), and 17.5% (95% CI 13.7 to 21.3), respectively (Table 2*A*). Assay performance was similar when pooling negative and indeterminate results for the total population (Table 2*B*). Among the letermovir and valganciclovir arms, assay performance was similar, with the exception of sensitivity at the 28-week timepoint posttransplant (Table 3). These findings were comparable in the sensitivity analysis for committee-confirmed CMV disease postprophylaxis (Supplementary Tables S6 and S7).

**Table 2.** QuantiFERON-CMV Assay Performance at Week 28 in the Total Population for (*A*) Positive Versus Negative Results and (*B*) Positive Versus Pooled Negative and Indeterminate Results for Prediction of Investigator-Reported Postprophylaxis CMV Disease by Week 52

A. Positive versus negative results in the total population			
QuantiFERON-CMV Result	Did Not Develop Postprophylaxis CMV Disease (n = 339)	Developed Postprophylaxis CMV Disease (n = 70)	PPV/NPV, % (95% CI)
Positive (n = 32)	28 (TP)	4 (FP)	PPV, 87.5 (76.0, 99.0)
Negative (n = 377)	311 (FN)	66 (TN)	NPV, 17.5 (13.7, 21.3)
**Sensitivity/specificity, % (95% CI)**	Sensitivity, 8.3 (5.3, 11.2)	Specificity, 94.3 (88.9, 99.7)	─

B. Positive versus pooled negative and indeterminate results in the total population			
QuantiFERON-CMV Result	Did NOT Develop Postprophylaxis CMV Disease (n = 376)	Developed Postprophylaxis CMV Disease (n = 84)	PPV/NPV, % (95% CI)
Positive (n = 32)	28 (TP)	4 (FP)	PPV, 87.5 (76.0, 99.0)
Negative and indeterminate (n = 428)	348 (FN)	80 (TN)	NPV, 18.7 (15.0, 22.4)
**Sensitivity/specificity, % (95% CI)**	Sensitivity, 7.4 (4.8, 10.1)	Specificity, 95.2 (90.7, 99.8)	─

95% CIs were calculated based on exact binomial tests.

$\mathrm{Sensitivity}=\;\frac{TP}{TP+FN}$ ; $\mathrm{Specificity}=\;\frac{TN}{TN+FP};\;\mathrm{PPV}=\;\frac{TP}{TP+FP}$; $\mathrm{NPV}\;=\;\frac{TN}{TN+FN}$.

Participants who developed CMV disease prior to week 28 were excluded.

Abbreviations: CMV, cytomegalovirus; FP, false positive; FN, false negative; NPV, negative predictive value; PPV, positive predictive value; TP, true positive; TN, true negative.

**Table 3.** QuantiFERON-CMV Assay Performance at Week 28 in the Letermovir and Valganciclovir Arms for Prediction of Investigator-Reported Postprophylaxis CMV Disease by Week 52

	Positive Versus Negative Results		Positive Versus Pooled Negative and Indeterminate Results	
	Letermovir	Valganciclovir	Letermovir	Valganciclovir
Sensitivity, % (95% CI)	2.4 (0.1, 4.8)	13.7 (8.6, 18.8)	2.2 (0.7, 4.4)	12.2 (7.7, 16.8)
Specificity, % (95% CI)	97.4 (92.5, 100.0)	90.3 (79.9, 100.0)	97.7 (93.3, 100.0)	92.5 (84.3, 100.0)
PPV, % (95% CI)	80.0 (45.0, 100.0)	88.9 (77.0, 100.0)	80.0 (45.0, 100.0)	88.9 (77.0, 100.0)
NPV, % (95% CI)	19.2 (13.7, 24.7)	15.6 (10.3, 21.0)	19.6 (14.4, 24.9)	17.7 (12.5, 22.9)

95% CIs were calculated based on exact binomial tests.

Participants who developed CMV disease prior to week 28 were excluded.

Abbreviations: CMV, cytomegalovirus; PPV, positive predictive value; NPV, negative predictive value.

### Week 52 QuantiFERON-CMV Result and Cytomegalovirus Events Through Week 52 Posttransplant

Approximately 81% (106/131) of participants with a positive QFT-CMV result at week 52 had a preceding CMV event (investigator-reported CMV disease and/or quantifiable CMV DNAemia) by week 52 compared to 23.7% (66/278) with a negative QFT-CMV result at week 52 (Supplementary Table S8).

## DISCUSSION

In this large, multicenter trial of CMV D+R− KTRs who received CMV prophylaxis with letermovir or valganciclovir for 28 weeks, CMV-CMI by QFT-CMV increased over time posttransplant, but had poor clinical utility for predicting postprophylaxis CMV disease.

There was progressively increasing QFT-CMV positivity over time posttransplant, indicating the acquisition of CMV-specific cellular immunity, which may be associated with reductions in immunosuppression during this time, as found by others [17, 25, 26]. The evolution of CMV-CMI by QFT-CMV in the letermovir and valganciclovir study arms was similar except for a slightly lower positivity rate in the letermovir arm at week 28. The association of letermovir prophylaxis with delayed CMV-CMI has been described in another study [30], suggesting that viral exposure is ultimately required to facilitate the development of CMV-CMI [31]. A post hoc analysis from the phase 3 trial showed that adherence was lower, breakthrough quantifiable CMV DNAemia occurred more frequently, and a greater number of participants discontinued prophylaxis in the valganciclovir arm compared to the letermovir arm [32]. We hypothesize that breakthrough CMV DNAemia and adverse events resulting in premature discontinuation of valganciclovir led to a higher rate of CMV DNAemia during the prophylaxis period, which may have contributed to the higher rate of positive QFT-CMV results in the valganciclovir arm at week 28. By week 52, nearly 30% of participants had a positive QFT-CMV result. There was a higher proportion of participants with preceding CMV events among those with a positive QFT-CMV compared to a negative QFT-CMV at week 52. However, there was also discordance between preceding CMV events and QFT-CMV results at week 52 in some of the participants, as shown in Supplementary Table S8.

The poor clinical utility of the QFT-CMV assay was driven by several key findings. First, the majority of participants with a negative QFT-CMV result at week 28 did not develop CMV disease by week 52 (82.5%). Second, only a small proportion of participants developed a positive QFT-CMV result at week 28 (∼8% in the overall population). Third, there was a relatively high incidence of CMV disease even among the participants with a positive result at week 28 (12.5%), and assay performance did not improve when pooling negative and indeterminate results. Although the PPV of the week 28 QFT-CMV result was relatively high (∼90%), the incidence of CMV disease among those with a positive result was not significantly lower than among those with a negative result (9.4% and 11.7%, respectively). For all these reasons, the results of QFT-CMV at the end of a standard 28-week prophylaxis period in CMV high-risk D+R− KTRs were not sufficiently clinically useful to guide decision-making for subsequent CMV monitoring or preventive strategies. This was evident for both the primary analysis for investigator-reported CMV disease, which provides greater generalizability by reflecting real-world clinical practice, and for the sensitivity analysis for committee-confirmed CMV disease based on specific diagnostic criteria [33].

Our findings are consistent with other studies of QFT-CMV in CMV D+R− SOT recipients [15–17, 21–26, 34]. However, our study includes a large sample size of participants from a controlled phase 3 trial that examined the evolution of CMV-CMI based on QFT-CMV results from a central laboratory and used the clinically relevant endpoint of CMV disease reported by investigators who were blinded both to the QFT-CMV results and trial intervention. This also allowed for a comparative assessment of QFT-CMV results with 2 different prophylaxis regimens, which was not possible in prior studies (Supplementary Table S1).

Other CMV-CMI assays (CMV-ELISPOT, T-Track) similarly appear to have limited clinical utility, specifically in the highest-risk CMV D+R− group of SOT recipients. One potential explanation may be that currently available assays do not assess other CMV-specific immune responses (humoral, NK-cell, etc.) that might also be relevant in protective immunity in this setting. Future studies that assess a larger breadth of CMV-specific immune responses have been suggested in a recent study [31].

Strengths of this study include that this is the largest, multicenter investigation of QFT-CMV in high-risk CMV D+R− KTRs collected at multiple prespecified timepoints in participants from a phase 3 clinical trial. Additional strengths include the performance of QFT-CMV at a central laboratory by blinded personnel, assessment of assay performance with pooled negative and indeterminate results, use of standardized CMV prophylaxis regimens, and use of the clinically relevant endpoint of CMV disease. A limitation was the infeasibility of assessing QFT-CMV performance at thresholds other than the manufacturer recommendations (cutoff for positivity of ≥0.2 IU/mL). But, the improvement in assay performance with lower thresholds in a prior study was modest [26]. The proportion of participants who received lymphocyte-depleting induction immunosuppression was nearly 50%, which is lower than rates in some transplant settings. However, we did not find an association between receipt of lymphocyte-depleting induction immunosuppression and the rate of QFT-CMV positivity at the end of the standard duration of prophylaxis in CMV D+R− KTRs in this study. Some participants had a positive QFT-CMV result in the absence of known preceding CMV DNAemia, while some had a negative QFT-CMV result despite preceding CMV DNAemia. Potential reasons for this include immune priming resulting from localized CMV replication within the allograft (without concomitant CMV DNAemia) and associated antigen presentation as shown previously [35] or “missed” CMV DNAemia that occurred between prespecified scheduled testing.

In summary, the results of the QFT-CMV assay assessed at the end of standard antiviral prophylaxis had limited clinical utility to predict postprophylaxis CMV disease in CMV D+ R− KTRs based on this study and should not be recommended for routine use. Given the important unmet need, other approaches, such as evaluating a larger breadth of CMV-specific adaptive immune responses (including humoral, T-cell, and NK-cell) or innate immune parameters, measuring CMV DNAemia in the donor kidney, or assessing other donor factors that may influence the development of CMV-CMI, might be useful and should be studied.

## Supplementary Material

ciaf632_Supplementary_Data

## Supplementary Data

Supplementary materials are available at *Clinical Infectious Diseases* online. Consisting of data provided by the authors to benefit the reader, the posted materials are not copyedited and are the sole responsibility of the authors, so questions or comments should be addressed to the corresponding author.
