# Supplementary material for: Cytomegalovirus (CMV)-Specific Cell-Mediated Immunity for Prediction of Postprophylaxis CMV Disease in a Phase 3 Trial of Letermovir Versus Valganciclovir Prophylaxis in Donor CMV-Seropositive/Recipient CMV-Seronegative Kidney Transplant Recipients
Source: Clin Infect Dis. 2025 Dec 1;82(2):e316–24. doi: 10.1093/cid/ciaf632 (PMC13016965; doi:10.1093/cid/ciaf632)
Supplement: ciaf632_Supplementary_Data [file ciaf632_supplementary_data.docx]

**SUPPLEMENT**

**Table S1.** Publications Assessing CMV-CMI in CMV D+R− KTRs

|  | **Prospective (Y/N)** | **Multicenter**  **(Y/N)** | **Type(s) of SOTs** | **Number of CMV D+R− KTRs** | **CMV-CMI Assay Performed at a Central Laboratory (Y/N, or NR)** | **Evaluated CMV Disease (as defined in the study) as an outcome (Y/N)** |
| --- | --- | --- | --- | --- | --- | --- |
| Limaye AP, et al. Clin Infect Dis 2025; | Y | Y | CMV D+R− kidney | 586 | Y (QFT-CMV) | Y |
| Solera JT, et al. Transplantation 2025; 109(3):527-35. | Y | N | CMV D+R−, R+ kidney, liver, heart, kidney-pancreas, kidney-liver | 27 | NR (QFT-CMV) | Y |
| Blom KB, et al. Front Immunol 2024; 15: 1414830. | Y | Y | CMV D+R−, D−R−, R+ kidney | 260 | Y (QFT-CMV) | N |
| Servais AM, et al. Transpl Infect Dis 2024; 26(4): e14291. | N | N | CMV D+R−, D−R−, R+ kidney | 29 | NR (CMV-TCIP, Viracor) | Y |
| Manuel O, et al. Clin Infect Dis 2024; 78:312–323. | Y | Y | CMV D+R−, R+ kidney, liver | 164 KTRs (not specified how many were CMV D+R−) | N (ELISPOT, T-Track CMV, Mikrogen) | Y |
| Dioverti MV, et al. Clin Transplant 2023; 37(12): e15143. | Y | N | CMV D+R− kidney, liver, heart, kidney-pancreas, multiorgan | 14 (prophylaxis)  6 (treatment) | NR (QFT-CMV) | Y |
| Kumar D, et al. Am J Transplant 2019; 19(9): 2505-16. | Y | Y | CMV D+R−, R+ kidney | 257 | Y (ELISPOT, T‐SPOT.CMV, Oxford Immunotec) | Y |
| Tarasewicz A, et al. Transplant Proc 2016; 48(5): 1650-3. | Y | N | CMV D+R−, D−R+, D+R+, D−R− kidney | 9 | NR (QFT-CMV) | Y |
| Manuel O, et al. Clin Infect Dis 2013; 56(6): 817-24. | Y | Y | CMV D+R− kidney, kidney-pancreas, liver, lung, heart, other | 68 | Y^a^ (QFT-CMV) | Y |
| Kumar D, et al. Am J Transplant 2009; 9(5): 1214-22. | Y | N | CMV D+R−, D−R+, D+R+ lung, kidney, liver, kidney-pancreas, other | 32 KTRs (not specified how many were CMV D+R−) | NR (QFT-CMV) | Y |

^a^Batch testing in 3 centers.

NR, not reported.

**Table S2.** Interpretation of QuantiFERON-CMV assay results

| Nil (IU/mL) | CMV minus Nil (IU/mL) | Mitogen minus Nil (IU/mL)^a^ | QuantiFERON-CMV assay result | Interpretation |
| --- | --- | --- | --- | --- |
| ≤8.0 | ≥0.2 and ≥25% Nil | Any | Reactive^b^ | **POSITIVE** – CMV-CMI detected |
|  | <0.2 OR ≥0.2 and <25% Nil | ≥0.5 | Nonreactive | **NEGATIVE** – CMV-CMI not detected |
|  |  | <0.5 | Indeterminate^c^ | **INDETERMINATE** for CMV-CMI |
| >8.0^d^ | Any | Any |  |  |

^a^Responses to the Mitogen positive control (and occasionally CMV antigens) can be commonly outside the range of the microplate reader. This has no impact on test results.

^b^Where CMV infection is not suspected, initially reactive results can be confirmed by retesting the original plasma samples in duplicate in the QuantiFERON-CMV assay. If repeat testing of one or both replicates is positive, the individual should be considered test reactive.

^c^In clinical studies, an indeterminate result among solid organ transplant patients, where a donor is reactive for CMV but the Mitogen control was below 0.5 IU/mL, has been shown to be clinically relevant. Such patients have the highest risk of developing CMV.

^d^In clinical studies, less than 0.25% of subjects had interferon gamma levels of >8.0 IU/mL for the value.

Qiagen: QuantiFERON®-CMV ELISA Package Insert. Published 2019. Available at: <https://www.qiagen.com/at/resources/download.aspx?id=42713b7e-2b8f-48b6-8198-44104d14aacb&lang=en>. Note: Table has been modified from the reference.

CMV, cytomegalovirus; CMV-CMI, CMV-specific cell-mediated immunity.

**Table S3.** Use of lymphocyte-depleting induction immunosuppression in participants with a negative, indeterminate, or positive QuantiFERON-CMV result at baseline

| Use of lymphocyte-depleting induction immunosuppression^a^ | Indeterminate result | | | Negative result | | | Positive result | | |
| --- | --- | --- | --- | --- | --- | --- | --- | --- | --- |
|  | LET  (n=153) | VGCV (n=150) | Total  (n=303) | LET  (n=127) | VGCV (n=135) | Total  (n=262) | LET  (n=3) | VGCV (n=4) | Total (n=7) |
| Yes | 100 (65.4) | 104 (69.3) | 204 (67.3) | 27 (21.3) | 27 (20.0) | 54 (20.6) | 1 (33.3) | 1 (25.0) | 2 (28.6) |
| No | 53 (34.6) | 46 (30.7) | 99 (32.7) | 100 (78.7) | 108 (80.0) | 208 (79.4) | 2 (66.7) | 3 (75.0) | 5 (71.4) |

Data shown as n (%).

^a^Receipt of ≥1 of the following lymphocyte-depleting agent(s) at the time of transplant: horse-derived or rabbit-derived antithymocyte globulin, alemtuzumab, or muromonab CD3.

LET, letermovir; VGCG, valganciclovir.

**Table S4.** Baseline demographics and characteristics of participants with and without a QuantiFERON-CMV result at week 28

| Demographic/  Characteristic | With a QuantiFERON result | | | Without a QuantiFERON result | | |
| --- | --- | --- | --- | --- | --- | --- |
|  | LET  (n=225) | VGCV  (n=242) | Total  (n=467) | LET  (n=64) | VGCV  (n=55) | Total  (n=119) |
| Male | 164 (72.9) | 168 (69.4) | 332 (71.1) | 46 (71.9) | 41 (74.5) | 87 (73.1) |
| Mean age in years (SD) | 49.7 (14.4) | 48.8 (15.3) | 49.2 (14.8) | 49.1 (15.5) | 53.6 (13.7) | 51.2 (14.8) |
| Race |  |  |  |  |  |  |
| Asian | 3 (1.3) | 9 (3.7) | 12 (2.6) | 1 (1.6) | 1 (1.8) | 2 (1.7) |
| Black or African American | 12 (5.3) | 23 (9.5) | 35 (7.5) | 9 (14.1) | 10 (18.2) | 19 (16.0) |
| White | 199 (88.4) | 203 (83.9) | 402 (86.1) | 51 (79.7) | 40 (72.7) | 91 (76.5) |
| Other | 11 (4.9) | 7 (2.9) | 18 (3.9) | 3 (4.7) | 4 (7.3) | 7 (5.9) |
| Use of lymphocyte-depleting induction immunosuppression^a^ | 93 (41.3) | 107 (44.2) | 200 (42.8) | 38 (59.4) | 31 (56.4) | 69 (58.0) |
| Donor type |  |  |  |  |  |  |
| Living | 92 (40.9) | 115 (37.2) | 182 (39.0) | 28 (43.8) | 25 (45.5) | 53 (44.5) |
| Deceased | 133 (59.1) | 152 (62.8) | 285 (61.0) | 36 (56.3) | 30 (54.5) | 66 (55.5) |

Data shown as n (%).

^a^Stratified by receipt of ≥1 of the following lymphocyte-depleting agent(s) at the time of transplant: horse-derived or rabbit-derived antithymocyte globulin, alemtuzumab, or muromonab CD3.

**Table S5.** Baseline demographics and characteristics of participants with an indeterminate, negative, or positive QuantiFERON-CMV result in the total population at week 28

|  | QuantiFERON-CMV result at week 28 | | |
| --- | --- | --- | --- |
| Demographic/  characteristic | Indeterminate result | Negative  result | Positive  result |
|  | (n=54) | (n=377) | (n=36) |
| Male | 34 (63.0) | 272 (72.1) | 26 (72.2) |
| Mean age in years (SD) | 48.2 (14.4) | 49.2 (14.9) | 50.4 (15.2) |
| Race |  |  |  |
| Asian | 1 (1.9) | 11 (2.9) | 0 (0.0) |
| Black or African  American | 9 (16.7) | 24 (6.4) | 2 (5.6) |
| White | 44 (81.5) | 325 (86.2) | 33 (91.7) |
| Other | 0 (0.0) | 17 (4.5) | 1 (2.8) |
| Use of lymphocyte-depleting induction immunosuppression^a^ | 24 (44.4) | 163 (43.2) | 13 (36.1) |
| Donor type |  |  |  |
| Living | 18 (33.3) | 152 (40.3) | 12 (33.3) |
| Deceased | 36 (66.7) | 225 (59.7) | 24 (66.7) |

Data shown as n (%).

^a^Stratified by receipt of ≥1 of the following lymphocyte-depleting agent(s) at the time of transplant: horse-derived or rabbit-derived antithymocyte globulin, alemtuzumab, or muromonab CD3.

**Table S6.** QuantiFERON-CMV assay performance at week 28 in the total population for A.) positive versus negative results and B.) positive versus pooled negative and indeterminate results for prediction of committee-confirmed post-prophylaxis CMV disease by week 52

A. Positive versus negative results in the total population

| QuantiFERON-CMV result | Did NOT develop post-prophylaxis CMV disease  (n=362) | Developed  post-prophylaxis  CMV disease  (n=47) | **PPV/NPV, %**  **(95% CI)** |
| --- | --- | --- | --- |
| Positive (n=32) | 29 (TP) | 3 (FP) | PPV, 90.6  (80.5, 100.0) |
| Negative (n=377) | 333 (FN) | 44 (TN) | NPV, 11.7  (8.4, 14.9 ) |
| **Sensitivity/Specificity, %**  **(95% CI)** | Sensitivity, 8.0  (5.2, 10.8) | Specificity, 93.6  (86.6, 100.0) | ─ |

B. Positive versus pooled negative and indeterminate results in the total population

| QuantiFERON-CMV result | Did NOT develop post-prophylaxis CMV disease  (n=409) | Developed  post-prophylaxis  CMV disease  (n=54) | **PPV/NPV, %**  **(95% CI)** |
| --- | --- | --- | --- |
| Positive (n=32) | 29 (TP) | 3 (FP) | PPV, 90.6  (80.5, 100.00) |
| Negative & indeterminate (n=431) | 380 (FN) | 51 (TN) | NPV, 11.8  (8.8, 14.9) |
| **Sensitivity/Specificity, %**  **(95% CI)** | Sensitivity, 7.1  (4.6,9.6) | Specificity, 94.4  (88.3, 100.00) |  |

95% confidence intervals (CI) were calculated based on exact binomial test.

Participants who developed CMV disease prior to week 28 were excluded.

Sensitivity = $\frac{TP}{TP+FN}$; Specificity = $\frac{TN}{TN+FP};$PPV = $\frac{TP}{TP+FP}$; NPV = $\frac{TN}{TN+FN}$.

FP, false positive; FN, false negative; TP, true positive; TN, true negative.

**Table S7.** QuantiFERON-CMV assay performance at week 28 in the letermovir and valganciclovir arms for prediction of committee-confirmed post-prophylaxis CMV disease by week 52

|  | Positive versus Negative Results | | | Positive versus Pooled Negative and Indeterminate Results | | |
| --- | --- | --- | --- | --- | --- | --- |
|  | Letermovir  % (95% CI) | Valganciclovir  % (95% CI) | | Letermovir  % (95% CI) | Valganciclovir  % (95% CI) | |
| Sensitivity, %  (95% CI) | 2.2 (0.1, 4.0) | | 13.7 (8.7, 18.6) | 2.0 (0.1, 4.0) | | 11.8 (7.5, 16.2) |
| Specificity, %  (95% CI) | 95.8 (87.8, 100.0) | | 91.3 (79.8, 100.0) | 96.3 (89.2, 100.0) | | 92.6 (82.7, 100.0) |
| PPV, %  (95% CI) | 80.0 (44.5, 100.0) | | 92.6 (82.7, 100.0) | 80.0 (45.0, 100.0) | | 92.6 (82.7, 100.0) |
| NPV, %  (95% CI) | 11.6 (7.2, 16.1) | | 11.7 (7.0, 16.5) | 11.8 (7.6, 16.1) | | 11.8 (7.5, 16.2) |

95% confidence intervals (CI) were calculated based on exact binomial test.

Participants who developed CMV disease prior to week 28 were excluded.

PPV, positive predictive value; NPV, negative predictive value.

**Table S8.** QuantiFERON-CMV assay result at week 52 in the total population for A.) positive versus negative results and B.) positive versus pooled negative and indeterminate results and CMV event from day 1 through week 52

A. Positive versus negative QFT-CMV results in the total population

| CMV event by week 52 | QuantiFERON-CMV result at week 52 | |
| --- | --- | --- |
|  | Positive (n=131) | Negative (n=278) |
| Developed post-prophylaxis CMV disease (n=80) | 47 | 33 |
| Did NOT develop post-prophylaxis CMV disease (n=329) | 84 | 245 |
| Had qCMV DNAemia (n=165) | 103 | 62 |
| Did NOT have qCMV DNAemia (n=244) | 28 | 216 |
| Developed post-prophylaxis CMV disease AND/OR qCMV DNAemia (n=172) | 106 | 66 |
| Did NOT develop post-prophylaxis CMV disease and NO qCMV DNAemia (n=237) | 25 | 212 |

B. Positive versus pooled negative and indeterminate QFT-CMV results in the total population

| CMV event by week 52 | QuantiFERON-CMV result at week 52 | |
| --- | --- | --- |
|  | Positive (n=131) | Negative & Indeterminate (n=322) |
| Developed post-prophylaxis CMV disease (n=91) | 47 | 44 |
| Did NOT develop post-prophylaxis CMV disease (n=362) | 84 | 278 |
| Had qCMV DNAemia (n=185) | 103 | 82 |
| Did NOT have qCMV DNAemia (n=268) | 28 | 240 |
| Developed post-prophylaxis CMV disease AND/OR qCMV DNAemia (n=192) | 106 | 86 |
| Did NOT develop post-prophylaxis CMV disease and NO qCMV DNAemia (n=261) | 25 | 236 |

Central CMV DNA testing. Investigator-reported CMV disease.

Participants who developed CMV disease prior to week 28 were included.

q, quantifiable.

**Figure S1. Point estimate for a positive versus a negative and indeterminate week 28 QuantiFERON-CMV stratified by baseline demographics and characteristics in the total population**

**
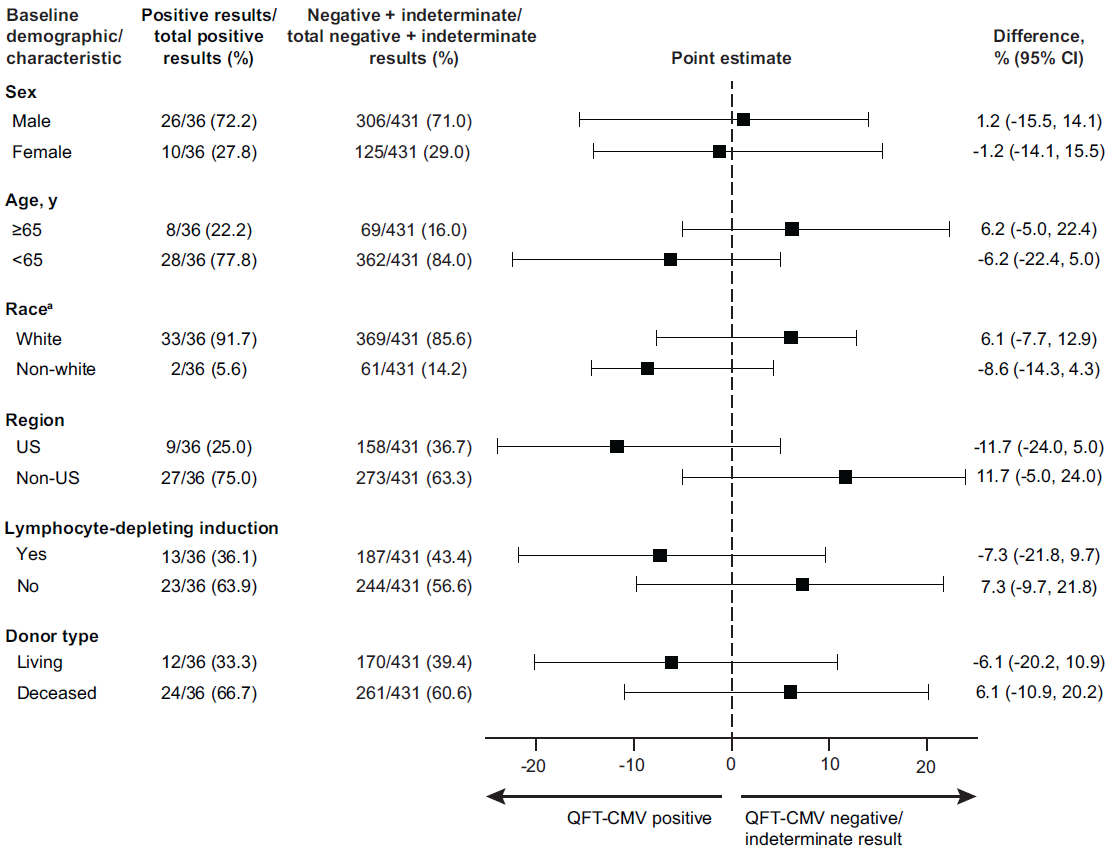
**

^a^Two participants did not report race.

**Figure S2. QuantiFERON-CMV assay results at week 28 in the total population and committee-confirmed post-prophylaxis CMV disease by week 52**

**
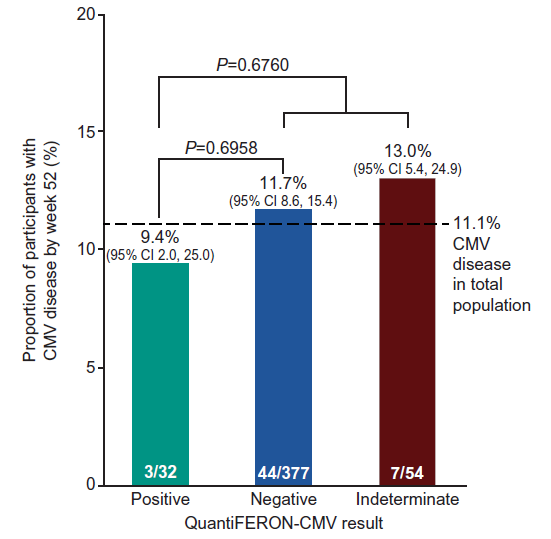
**

^a^Chi square test with two-tailed P-value for positive versus negative QuantiFERON-CMV results and positive versus pooled negative and indeterminate results.

**MK-8228-002 Investigational Team**

The authors thank the Clinical Adjudication Committee, the primary investigators, and the Data Monitoring Committee.

**Clinical Adjudication Committee**

- Camille N. Kotton (Chair), Massachusetts General Hospital, Boston, Massachusetts, USA
- Alison G. Freifeld, University of Nebraska Medical Center, Omaha, Nebraska, USA
- Per Ljungman, Karolinska University Hospital, Stockholm, Sweden
- David R. Snydman, Tufts Medical Center, Boston, Massachusetts, USA

**Primary Investigators**

| **Name*** | **Site** | **Location** |
| --- | --- | --- |
| Laura Alicia Barcan | Hospital Italiano de Buenos Aires | CABA, CABA, Argentina |
| Maria del Carmen Rial | Instituto de Nefrologia Nephrology S.A. | Buenos Aires, CABA, Argentina |
| Judith Gaite | Clinica de Nefrologia Urologia y Enfermedades Cardiovasculares | Santa Fe, Argentina |
| Sandra Lambert | Hospital El Cruce Nestor Carlos Kirchner | Buenos Aires, Argentina |
| Elena Rosa Temporiti | CEMIC | Buenos Aires, Argentina |
| Marcela Silvia Maurich | Instituto de Cardiología de Corrientes Juana F. Cabral | Corrientes, Corrientes, Argentina |
| Katherine Barraclough/Jean Tan | Royal Melbourne Hospital | Parkville, Victoria, Australia |
| Scott Campbell | Princess Alexandra Hospital | Woolloongabba, Queensland, Australia |
| Jeremy Chapman/Philip O’Connell/  Germaine Wong | Westmead Hospital | Westmead, New South Wales, Australia |
| William Mulley | Monash Health-Monash Medical Centre | Clayton, Victoria, Australia |
| Graeme Russ/Robert Peter Carroll | Royal Adelaide Hospital | Adelaide, South Australia, Australia |
| Kate Wyburn | Royal Prince Alfred Hospital | Camperdown, New South Wales, Australia |
| Gabriela Berlakovich | Medizinische Universität Wien | Wien, Wien, Austria |
| Stefan Schneeberger | Medizinische Universitat Innsbruck | Innsbruck, Tirol, Austria |
| Rachel Hellemans | Universitair Ziekenhuis Antwerpen | Edegem, Antwerpen, Belgium |
| Jean-Michel Hougardy/Emine Nilufer Broeders | Cliniques Universitaires de Bruxelles - CUB - Hopital Erasme | Bruxelles, Bruxelles-Capitale, Region de, Belgium |
| Dirk Kuypers | UZ Leuven - Campus Gasthuisberg | Leuven, Vlaams-Brabant, Belgium |
| Karen Doucette | University of Alberta Hospital | Edmonton, Alberta, Canada |
| Simon Dufresne | Hopital Maisonneuve-Rosemont CIUSSS de l Est de L Ile de Montreal | Montreal, Quebec, Canada |
| John Gill | St. Paul's Hospital | Vancouver, British Columbia, Canada |
| Atul Humar | Toronto General Hospital | Toronto, Ontario, Canada |
| James Lan | Vancouver General Hospital | Vancouver, British Columbia, Canada |
| Jairo Camilo Montero | Clinica Colsanitas S.A. Sede Clinica Universitaria Colombia | Bogota, Distrito Capital de Bogota, Colombia |
| Rodolfo Eduardo Torres Serrano | Sociedad de Cirugia de Bogota Hospital de San Jose | Bogota, Distrito Capital de Bogota, Colombia |
| Adalberto Pena | Fundacion Cardiovascular de Colombia | Bucaramanca, Santander, Colombia |
| Luis Valderrama | Centro Medico Imbanaco de Cali S.A | Cali, Valle del Cauca, Colombia |
| Laetitia Albano | Hopital Pasteur | Nice, Alpes-Maritimes, France |
| Antoine Durrbach/Severine Beaudreuil | CHU - Hopital de Bicetre | Le kremlin bicetre, Val-de-Marne, France |
| Matthias Buchler | C.H.R.U Bretonneau | Tours, Indre-et-Loire, France |
| Alexandre Hertig/Francois Helene | Hopital Tenon | Paris, Paris, France |
| Nassim Kamar | CHU Rangueil | Toulouse, Haute Garonne, France |
| Pierre Merville | CHU de Bordeaux. Hopital Pellegrin | Bordeaux, Gironde, France |
| Klemens Budde | Charite Universitaetsmedizin Berlin | Berlin, Berlin, Germany |
| Hermann Haller | Medizinische Hochschule Hannover | Hannover, Niedersachsen,  Germany |
| Oliver Witzke | Universitaetsklinikum Essen | Essen, Nordrhein-Westfalen, Germany |
| Gyoergy Lazar | Szegedi Tudomanyegyetem | Szeged, Csongrad, Hungary |
| Zoltan Mathe/Adam Remport | Semmelweis Egyetem | Budapest, Budapest, Hungary |
| Balazs Nemes | Debreceni Egyetem | Debrecen, Debrecen,  Hungary |
| Luigi Biancone | A.O.U. Citta della Salute e della Scienza di Torino | Torino, Piemonte, Italy |
| Franco Citterio | Policlinico Gemelli Instituto di Clinica Chirurgica | Rome, Lazio, Italy |
| Paolo Rigotti | Azienda Ospedaliera di Padova U.O.C. Trapianti Rene e Pancreas | Padova, Veneto, Italy |
| Antonio Secchi | IRCCS Ospedale San Raffaele di Milano | Milano, Milano, Italy |
| Guillermo Antonio Mondragon Ramirez | Instituto Mexicano de Trasplantes S C | Cuernavaca, Morelos,  Mexico |
| Luis Eduardo Morales | Instituto Nacional de Ciencias Medicas y Nutricion Salvador Zubiran | Mexico City, Distrito Federal, Mexico |
| Eduardo Mancilla Urrea | Instituto Nacional de Cardiologia Ignacio Chavez | Mexico City, Distrito Federal, Mexico |
| Gustavo Martinez | Faicic S de RL de CV | Veracruz, Veracruz, Mexico |
| Rafael Reyes Acevedo | Centenario Hospital Miguel Hidalgo | Aguascalientes, Aguascalientes, Mexico |
| Ian Dittmer | Auckland City Hospital | Auckland, Auckland, New Zealand |
| Maciej Glyda | Szpital Wojewodzki w Poznaniu | Gdansk, Pomorskie, Poland |
| Marta Crespo Barrio | Hospital del Mar | Barcelona, Barcelona, Spain |
| Amado de Andres Belmonte | Hospital Doce de Octubre | Madrid, Madrid, Spain |
| Fritz Diekmann | Hospital Clinic i Provincial de Barcelona | Barcelona, Barcelona, Spain |
| Alex Gutierrez Dalmau | Hospital Universitario Miguel Servet | Zaragoza, Zaragoza, Spain |
| Josep Maria Cruzado Garrit | Hospital Universitari de Bellvitge IDIBELL | L Hospitalet de Llobregat, Barcelona, Spain |
| Francesc Moreso Mateos | Hospital Universitari Vall de Hebron | Barcelona, Barcelona, Spain |
| Joyce Popoola | St Georges University Hospitals NHS Foundation Trust | Tooting, London, UK |
| Robin K. Avery | Johns Hopkins Hospital | Baltimore, Maryland, USA |
| John W. Baddley/Nathan Erdmann | UAB | Birmingham, Alabama, USA |
| Emily Blumberg | University of Pennsylvania | Philadelphia, Pennsylvania, USA |
| Suphamai Bunnapradist | UCLA Medical Center | Los Angeles, California, USA |
| Stuart H. Cohen | UC Davis Medical Center | Sacramento, California, USA |
| Alejandro Diez | The Ohio State University Wexner Medical Center | Columbus, Ohio, USA |
| Diana F. Florescu  (Coordinating Investigator) | University of Nebraska Medical Center | Omaha, Nebraska, USA |
| Julia Garcia-Diaz | Ochsner Clinic Foundation | New Orleans, Louisiana, USA |
| Jennifer Husson | University of Maryland Medical Center | Baltimore, Maryland, USA |
| Nicolas Issa | Brigham & Women's Hospital | Boston, Massachusetts, USA |
| Dean Kim | Henry Ford Hospital | Detroit, Michigan, USA |
| Stuart Knechtle | Duke University Medical Center | Durham, North Carolina, USA |
| Dhiren Kumar | Virginia Commonwealth University | Richmond, Virginia, USA |
| Ajit P. Limaye | University of Washington Medical Center | Seattle, Washington, USA |
| Anup Magan Patel | Saint Barnabas Medical Center | Livingston, New Jersey, USA |
| Kathleen M. Mullane/Jennifer Christoff Pisano | University of Chicago | Chicago, Illinois, USA |
| Minh-Hong Nguyen | University of Pittsburgh | Pittsburgh, Pennsylvania, USA |
| Marcus Pereira | Columbia University Medical Center | New York, New York, USA |
| Meenakshi Rana | Icahn School of Medicine at Mount Sinai | New York, New York, USA |
| Vinayak Rohan | Medical University of South Carolina | Charleston, South Carolina, USA |
| Gowri Satyanarayana | Vanderbilt University Medical Center | Nashville, Tennessee, USA |
| Catherine B. Small | New York Presbyterian Hospital - Weill Cornell Medical Center | New York, New York, USA |
| Robert J. Stratta | Wake Forest University Baptist Medical Center | Winston Salem, North Carolina, USA |
| Tim E. Taber/Asif Sharfuddin | Indiana University | Indianapolis, Indiana, USA |
| Flavio Vincenti | University of California-San Francisco | San Francisco, California, USA |
| Janice Wes Brown | Stanford Health Care | Stanford, California, USA |

*****Primary investigators who screened and/or randomized participants. If the primary investigator changed, the initial primary investigator's name is followed by a slash (/) and the new primary investigator's name.
